# Molecular Characterisation of Long-Acting Insulin Analogues in Comparison with Human Insulin, IGF-1 and Insulin X10

**DOI:** 10.1371/journal.pone.0034274

**Published:** 2012-05-08

**Authors:** Bo F. Hansen, Tine Glendorf, Anne C. Hegelund, Anders Lundby, Anne Lützen, Rita Slaaby, Carsten Enggaard Stidsen

**Affiliations:** Diabetes Research Unit, Novo Nordisk A/S, Måløv, Denmark; Postgraduate Medical Institute & Hull York Medical School, University of Hull, United Kingdom

## Abstract

**Aims/Hypothesis:**

There is controversy with respect to molecular characteristics of insulin analogues. We report a series of experiments forming a comprehensive characterisation of the long acting insulin analogues, glargine and detemir, in comparison with human insulin, IGF-1, and the super-mitogenic insulin, X10.

**Methods:**

We measured binding of ligands to membrane-bound and solubilised receptors, receptor activation and mitogenicity in a number of cell types.

**Results:**

Detemir and glargine each displayed a balanced affinity for insulin receptor (IR) isoforms A and B. This was also true for X10, whereas IGF-1 had a higher affinity for IR-A than IR-B. X10 and glargine both exhibited a higher relative IGF-1R than IR binding affinity, whereas detemir displayed an IGF-1R:IR binding ratio of ≤1. Ligands with high relative IGF-1R affinity also had high affinity for IR/IGF-1R hybrid receptors. In general, the relative binding affinities of the analogues were reflected in their ability to phosphorylate the IR and IGF-1R. Detailed analysis revealed that X10, in contrast to the other ligands, seemed to evoke a preferential phosphorylation of juxtamembrane and kinase domain phosphorylation sites of the IR. Sustained phosphorylation was only observed from the IR after stimulation with X10, and after stimulation with IGF-1 from the IGF-1R. Both X10 and glargine showed an increased mitogenic potency compared to human insulin in cells expressing many IGF-1Rs, whereas only X10 showed increased mitogenicity in cells expressing many IRs.

**Conclusions:**

Detailed analysis of receptor binding, activation and *in vitro* mitogenicity indicated no molecular safety concern with detemir.

## Introduction

Increased interest in molecular safety of insulin analogues was stimulated by four epidemiological studies in this Journal in June 2009 [1]–[4], three of which suggested an association between the use of insulin glargine (glargine) and cancer [1]–[3]. A subsequent case-control study also suggested an association between glargine and an increased cancer risk, although this finding was restricted to high doses of glargine (≥3IU/kg/day) [5]. These studies have not been without criticism [6] and, unfortunately, at present the available randomised controlled trials (RCTs) are of quite limited size [7]. In addition, traditional animal toxicological studies with long-acting insulin analogues have been restricted to limited dose ranges due to death from hypoglycemia at escalated doses. Therefore, emphasis has now been put on the molecular characteristics of insulin analogues during safety evaluation.

**Figure 1:**
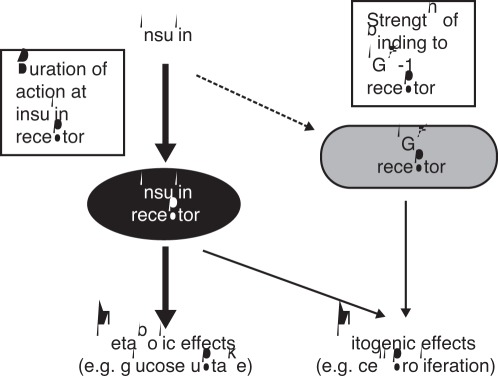
Potential mechanisms influencing the balance of metabolic and mitogenic actions of insulin-like molecules. Reprinted with kind permission from Springer Science & Business Media: Hansen *et al.* [9], Fig. 2. IGF-1, insulin-like growth factor 1.

The potential for modified insulin molecules to possess increased mitogenic potencies relative to human insulin has been recognised ever since a prototype rapid-acting analogue, insulin X10 (B10Asp), was found to dose-dependently increase the incidence of mammary tumours in female Sprague-Dawley rats [8], [9]. Subsequent investigations showed this analogue to have increased affinity for the IGF-1 receptor (insulin-like growth factor 1) (IGF-1R) relative to the insulin receptor (IR), in contrast to human insulin and other analogues not showing increased mitogenicity [10]–[13]. In addition, insulin X10 (X10) had increased residence time at the IR, eliciting prolonged IR activation [10], [14]. Each of these properties represents a feasible mechanism by which X10 could evoke an increased mitogenic response compared to human insulin (Fig. 1) [7], [9].

Despite renewed investigations into the molecular safety characteristics of insulin analogues, studies have produced conflicting results [15]. In order to clarify some of the remaining uncertainties and resolve some of the inconsistencies from earlier research, we have undertaken a comprehensive series of experiments employing robust laboratory methodologies. In this study, we have systematically investigated the properties of long acting insulin analogues with regard to receptor binding, receptor activation, duration of receptor activation in cells expressing IR-A, IR-B or IGF-1R, and mitogenic potency in two different cell types. Human insulin was included as the reference control, and the known mitogens IGF-1 and X10 as positive controls. In order to perform these experiments in a meaningful way allowing comparison among different ligands it is critical to perform full-dose response curves [7]. It is equally critical to use cells of identical age/life-cycle to obtain an adequate mitogenic response [16].

## Methods

### Materials

Human insulin, insulin detemir (detemir), glargine, X10 and IGF-1 were produced by recombinant DNA techniques and purified at Novo Nordisk A/S (Diabetes Research Unit, Måløv, Denmark). Long R3 IGF-1 (LR3-IGF-1, Sigma-Aldrich, Steinheim, Germany) was employed instead of IGF-1 for stimulation of IGF-1 receptor activity in order to avoid confounding effects from IGF binding proteins. ^125^I-labelled human insulin and ^125^I-labelled human IGF-1 were prepared at Novo Nordisk A/S (CMC Supply, Måløv, Denmark). For binding assays, human IR and human IGF-1R were semipurified either by homogenisation and centrifugation (membrane-associated receptors) or by wheat germ agglutinin (solubilised receptors) from baby hamster kidney (BHK) cells stably transfected with the pZem219B vector containing the human IR-A, IR-B or IGF-1R cDNAs alone or in combination [17]. Other chemicals were of reagent grade. The IR-specific antibody 83–7 and the IGF-1R-specific antibody 24–31 were licensed from Professor K. Siddle, University of Cambridge, UK [18], [19].

### Receptor Number

Receptors were quantified using QIFIKIT (Dako, Denmark) according to manufacturer’s protocols using either the murine monoclonal antibody 83–7 against the human IR, 24–31 against the human IGF-1R, or an isotype control antibody. Cells were analysed using an LSRFortessa (BD Biosciences, Franklin Lakes, NJ, USA).

### Receptor Binding

The relative binding affinities of the study ligands for the two IR isoforms and for the IGF-1R were measured using both solubilised and membrane-associated receptor systems.

#### Receptor binding assays (solubilised receptors)

The relative binding affinities of the study ligands for either solubilised human IR-A or IR-B were determined by competition binding in a scintillation proximity assay (SPA) setup as previously published [20]. In brief, dilution series in quadruplicate of human insulin and insulin analogues were performed in 96-well Optiplates (Perkin-Elmer Life Sciences, Boston, MA, USA) followed by the addition of SPA beads (Anti-Mouse polyvinyltoluene [PVT] SPA Beads, GE Healthcare, Waukesha, WI, USA), anti-IR mouse antibody 83–7, solubilised IR-A or IR-B, and [^125^I-TyrA14]-human insulin in a binding buffer consisting of 100 mM HEPES (pH 7.8), 100 mM NaCl, 10 mM MgSO_4_, and 0.025% (v/v) Tween 20 (Tween 20 was added to avoid adhesion of insulin to the assay plate). Increasing concentrations of human insulin or insulin analogue was used, typically between 0.001 and 30 nM. Plates were incubated with gentle shaking for 24 h at 22°C, centrifuged at 2000 rpm for 2 minutes and counted on a TopCount NXT (Perkin-Elmer Life Sciences). IGF-1R assays were conducted essentially as for the IR binding assays except that solubilised IGF-1 receptors and [^125^I-Tyr31]-human IGF-1 were employed.

#### Receptor binding assays (membrane-associated receptors)

The relative binding affinities of the different ligands for either the membrane-associated human IR-A or IR-B were determined by competition binding in a SPA setup. Assays were performed in duplicate in 96-well OptiPlates (Perkin-Elmer Life Sciences). Membrane protein was incubated with gentle agitation for 150 minutes at 25°C with 50 pM [^125^I-TyrA14]-human insulin in a total volume of 200 µl assay buffer (50 mM HEPES, 150 mM NaCl, 5 mM MgSO_4_, 0.01% Triton X−100, 0.1% ovalbumin, Complete EDTA-free protease inhibitors), 50 µg of wheat germ agglutinate (WGA)-coated PVT microspheres (GE Healthcare) and increasing concentrations of ligand (typically between 0.01 and 1,000 nM). The assays were terminated by centrifugation of the plate at 2000 rpm for 2 minutes and bound radioactivity quantified by counting in a TopCount NXT (Perkin-Elmer Life Sciences). IGF-1R assays were conducted essentially as for the IR binding assays except that membrane-associated IGF-1 receptors and 50 pM [^125^I-Tyr31]-human IGF-1 were employed.

### Hybrid Receptor Binding

WGA purification of solubilised receptors: cells were lysed in 50 mM HEPES pH 8.0, 150 mM NaCl, 1% Triton X−100, 2 mM EDTA, 10% glycerol. The cleared cell lysate was batch absorbed with WGA-agarose (Lectin from Triticum vulgaris-Agarose, L1394, Sigma-Aldrich) for 90 minutes. After 20 volumes of washes with 50 mM HEPES pH 8.0, 150 mM NaCl, 0.1% Triton X−100, the receptors were eluted with 50 mM HEPES, pH 8.0, 150 mM NaCl, 0.1% Triton X−100, 0.5 M N-acetyl glucosamine, 10% glycerol. All buffers contained Complete (Roche Diagnostic GmbH, Mannheim, Germany).

SPA for hybrid receptors: WGA- purified hybrid receptors of both isoforms of IR were used. SPA PVT anti-mouse beads (Perkin-Elmer Life Sciences) were incubated with IR antibody 83–7 and hybrid receptors for five hours at room temperature. The SPA beads were washed twice with buffer to remove homodimer IGF-1R and any other receptors not bound to the SPA beads, and ^125^I-IGF-1 was added. Dilution series of ligands were prepared in a Packard Optiplate 96 and the SPA beads added. The final concentration of ^125^I-IGF-1 was 5000 cpm/200 µl and the buffer composition was 100 mM HEPES, pH 7.8, 100 mM NaCl, 10 mM MgSO_4_, 0.025% Tween-20. The plate was rocked gently for 18 hours at room temperature, centrifuged and counted in a TopCounter. IC50 values were determined using non-linear regression algorithm in GraphPad Prism 5.0 (GraphPad Software Inc., San Diego, CA, USA).

### Receptor Activation/deactivation

#### IR activation

Activation of the two IR isoforms was assessed by the ability of the study ligands to phosphorylate three sites chosen from the three main regions of the IR beta unit, namely J (Juxtamembrane), K (Kinase) and C (C-peptide). The respective phosphorylation sites were (using IR-B terminology) 972 ( = 960 for IR-A), 1158 ( = 1146 for IR-A) and 1334 ( = 1322 for IR-A).

BHK cells overexpressing either IR-A, IR-B or IGF-1R were seeded in 12-well plates and grown until 90–100% confluence in DMEM (Gibco®, Invitrogen, Carlsbad, CA, USA) containing 10% FBS, 100 µg/ml penicillin and 100 U/ml streptomycin. Cells were stimulated with increasing concentrations of ligands (0–1000 nM) for 30 min in DMEM (Gibco®) medium containing 0.1% human serum albumin (HSA). Subsequently, cells were washed three times in ice-cold phosphate buffered saline (PBS) and snap-frozen by pouring liquid N_2_ into the wells. Cells were lysed in 100 µL lysis buffer (cell extraction buffer from BioSource, Invitrogen, Carlsbad, CA, USA; 1 mM AEBSF, and protease inhibitor cocktail from Sigma-Aldrich). Protein concentrations were measured with Pierce BCA Protein Assay Kit and equal amounts of protein loaded into Phospho-IR-ELISA wells (IR(pY972), IR(pY1158) and IR(pY1334) (Invitrogen). Phosphorylation of the three representative sites was measured according to the manufacturer’s protocol (Invitrogen).

#### IGF-1R Activation

Cells were grown as stated above and stimulated with ligands and lysates prepared as for IR activation. Lysates were analysed for IGF-1R activation by Western blotting using an anti-phospho-IGF-1R antibody (Ab5681 Abcam, Cambridge, UK) diluted 1∶1000 in Starting Block T20 Tris buffered saline (TBS) Blocking buffer (Thermo # 37543) and incubated overnight at 4°C with slight agitation. Subsequently, blots were incubated with secondary antibody (goat anti-rabbit IgG HRP 170–6515, Bio-Rad, California, USA) diluted 1∶3000 in Starting Block T20 (TBS) Blocking buffer (Thermo # 37543) and incubated for 1 hr at RT. Phosphorylated IGF-1R was visualised using SuperSignal West Pico Chemoluminescent Substrate (Thermo Scientific, Waltham, MA, USA) and band intensities quantified using a Fuji Imager LAS3000.

#### Duration of Activation

To evaluate the duration of signal after stimulation with ligands, cells were incubated for 30 min (10 and 30 nM [X10], 100 and 300 nM [human insulin and glargine], 1000 and 10,000 nM [detemir and IGF-1R]) in DMEM (Gibco®) medium containing 0.1% HSA, 100 µg/ml penicillin, 100 U/ml streptomycin and washed thoroughly three times in pre-warmed medium containing 0.1% HSA. Subsequently, cells were incubated for 0–5–10–20–30–45–60 min at 37°C whereupon phosphorylations of IR or IGF-1R were measured as described above. Phosphorylation at t = 0 was defined as 100%. Results were calculated as the average of the two applied concentrations for each ligand.

### Cell Mitogenicity

Mitogenicity of the study ligands was measured in two cell types: human mammary epithelial cells (HMEC) (obtained from Lonza, Basel, Switzerland, as cryopreserved cells at passage number 7), which express predominantly IGF-1R (∼21 times more IGF-1R than IR), and L6-myoblasts. The L6 muscle cells were obtained from ATCC and stably transfected with human insulin receptors to over-express human IR-A [16]. Thus, the L6-hIR cells express ∼200 times more IR compared to HMEC.

The HMECs were cultured in mammary epithelial growth medium (MEGM®) containing bovine insulin (5 µg/ml), bovine pituitary extract (50 µg/ml), hydrocortisone (0.5 µg/ml), epidermal growth factor (10 ng/ml) and gentamicin/amphotericin-B. Cells were passaged, at most, eight times covering approximately 5 weeks.

For mitogenicity experiments, cells were seeded at a density of 4×10^3^ cells/well in 96-well plates and incubated for 24 h in assay medium (mammary epithelial basal medium [MEBM®] containing bovine pituitary extract [50 µg/ml], hydrocortisone [0.5 µg/ml], epidermal growth factor [10 ng/ml] and gentamicin/amphotericin-B) after which dilution series of ligands were added. Plates were incubated for 72 h at which 0.125 µCi/well [3H]-thymidine was added at t  = 70 h. Cells were harvested using a cell harvester and scintillation liquid added to the dried filter plates after which radioactivity was counted in a TopCount NXT (all from Perkin-Elmer Life Sciences). L6-hIR cells were cultured in growth medium consisting of DMEM, 10% bovine serum, 100 U/ml penicillin, 100 µg/ml streptomycin, 2 mM glutamine, 1 mg/ml Geneticin (all from Gibco, Invitrogen), and 1 µM human insulin (Actrapid®, Novo Nordisk). Cells were passaged at most 30 times and subcultured every 2–3 days.

For mitogenicity experiments, L6-hIR cells were synchronised by topoinhibition (48 hrs) and serum starvation (24 hrs) prior to stimulation with test compounds [16].

Synchronised and starved L6-hIR cells were harvested and seeded at a density of 4×10^4^ cells/well in 96-well plates and incubated for 1 h in assay medium (DMEM, 0.1% FCS (foetal calf serum), 100 U/ml penicillin, 100 µg/ml streptomycin, 2 mM glutamine, 1 mg/ml Geneticin (all from Gibco, Invitrogen), after which dilution series of ligands were added. Plates were incubated for approximately 18 hrs at which 0.125 µCi/well [3H]-thymidine was added. After 2 hrs of incubation, cells were harvested using a cell harvester and scintillation liquid added to the dried filter plates after which radioactivity was counted in a TopCount NXT (all from Perkin-Elmer Life Sciences).

#### Data Analysis

In accordance with the European Pharmacopoeia [21], IR and IGF-1R receptor binding data were fitted using a four parameter sigmoidal algorithm developed for bioassays [22]. The binding affinities of the analogues were calculated relative to that of the human insulin standard [IC50(insulin)/IC50(analogue) × 100%] measured within the same plate. For stimulatory responses, the dose-response curves were fitted by non-linear regression using GraphPad Prism 5 (GraphPad Software Inc.) and potencies were calculated (if appropriate) relative to that of the human insulin standard [EC50 (insulin)/EC50 (analogue) × 100%].

## Results

### IR and IGF-1R Binding

**Figure 2:**
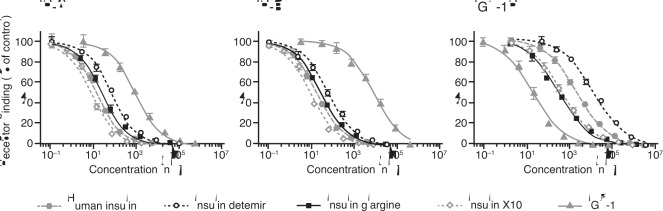
Dose-response curves for ligand binding of the insulin receptor isoforms A and B and the IGF-1 receptor. These were determined by competition binding in a scintillation proximity assay using solubilised receptors. Curves are representative; each data point is the mean +/− SEM of quadruplicate measurements. IR, insulin receptor; IGF-1, insulin-like growth factor 1; IGF-1R, insulin-like growth factor 1 receptor.

The relative binding affinities for the long-acting insulin analogues, X10 and IGF-1 are summarised in Table 1 and examples of full dose-response curves from competition binding experiments are presented in Fig. 2. The binding affinities of the insulin analogues for both the A and B isoform of the IR as well as the IGF-1R were determined using both solubilised and membrane-bound receptor systems. All the insulin analogues tested displayed a balanced IR-A to IR-B binding affinity ratio, whereas IGF-1 showed a higher IR-A than IR-B affinity. Detemir displays lower receptor binding affinities in the membrane-bound receptor systems compared to the solubilised receptor assay, reflecting the fact that ovalbumin is present in the binding assay with the membrane-bound, but not the solubilised receptors. Detemir exhibited both a decreased IR and IGF-1R affinity compared to human insulin and displayed a IGF-1R:IR affinity ratio of ≤1 relative to human insulin. Glargine bound to the IR with an affinity closer to that of human insulin, but showed a 7- to 10-fold increase in binding affinity for the IGF-1R relative to human insulin. X10 displayed a 2- to 3-fold increase for the IR, while the binding affinity for the IGF-1R was increased 4- to 5-fold. As expected, the IR binding affinity of IGF-1 was low compared to human insulin, whereas a large increase in IGF-1R affinity was observed.

**Table 1:** Relative binding affinities for insulin receptor isoform A and isoform B and IGF-1 receptors.

Solubilised receptors	(Affinity as % of human insulin)				Ratio relative to that of human insulin	
	IR-A	IR-B	IGF-1R		IR-A/IR-B	IGF-1R/IR
Human insulin	100	100		100	1	1
Insulin detemir	23±2	26±2	10±0.3		1	0.4
Insulin glargine	81±8	84±8	727 ±74		1	8.8
Insulin X10	268±27	299±41	480±31		1	1.7
IGF-1	1.3±0.1	0.1±0.01	17173±2270		11	13,000–170,000
**Membrane-bound receptors**	**(Affinity as % of human insulin)**				**Ratio relative to that of human insulin**	
	**IR-A**	**IR-B**	**IGF-1R**		**IR-A/IR-B**	**IGF-1R/IR**
Human insulin	100	100		100	1	1
Insulin detemir	6±1	5±1	4±1		1	0.8
Insulin glargine	70±12	63±13	1044±161		1	15.6
Insulin X10	265±47	213±26	428±37		1	1.8

Affinities were determined by insulin competition binding in a scintillation proximity assay; data are means (± SD) of quadruplicates (solubilised receptors) or duplicates (membrane-bound receptors).

### IR/IGF-1R Hybrid Receptor Binding

**Figure 3:**
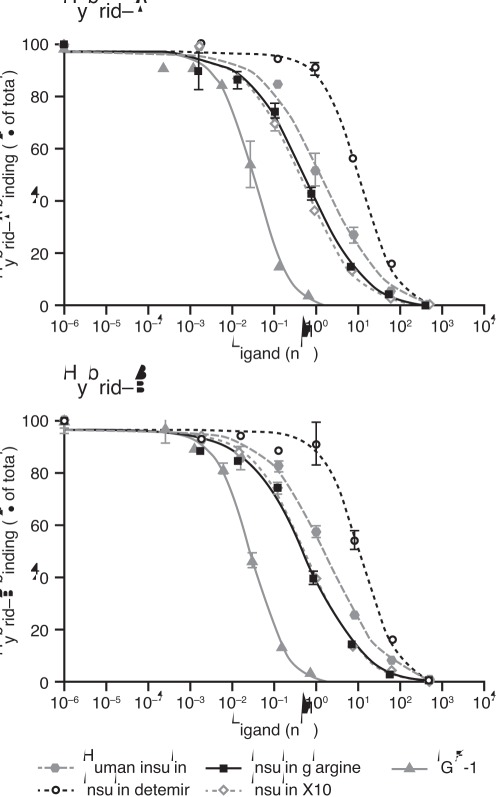
Competition curves for displacement of 125I-IGF-1 from Hybrid-A and Hybrid-B with human insulin IGF-1, insulin detemir, insulin glargine, insulin X10 or IGF-1 in SPA binding assay. The graphs are representatives of three experiments. Each point in the graphs is the mean (±SE) of three measurements. IGF-1, insulin-like growth factor 1; SPA, scintillation proximity assay.

Binding of human insulin, IGF-1, X10, glargine and detemir to hybrid receptors formed between IGF-1R and IR were studied (Table 2); both splice variants of the hybrid receptors were examined (Fig. 3). X10 and glargine bound with three-fold higher affinity than human insulin to hybrid receptors, whereas detemir-bound with a four-fold decreased affinity to hybrid receptors compared to human insulin. We did not observe any significant difference between the hybrid receptor splice variants for any of the analogues (Table 2).

**Table 2:** Relative binding affinities for Hybrid-A (IR-A/IGF-1R) and Hybrid-B (IR-B/IGF-1R).

Ligand binding relative to human insulin (%)					
Receptor	Human insulin	IGF-1	Insulin X10	Insulin detemir	Insulin glargine
Hybrid-A	100	6059±701	342±13	17±6	321±61
Hybrid-B	100	8243±2125	454±204	18±3	327±101

IC50 values were determined in scintillation proximity assays for displacement of ^125^I-IGF-1 from receptors with human insulin, IGF-1, insulin X10, insulin detemir or insulin glargine. Relative binding compared to human insulin binding is given in percent. Data represent mean (±SD) from three independent experiments.

IGF-1, insulin-like growth factor 1.

### Receptor Activation

**Figure 4:**
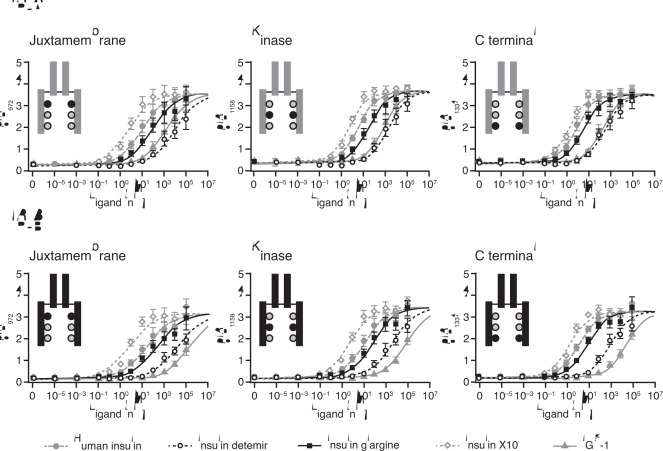
Dose-response curves for potencies for activation of insulin receptor isoform A and isoform B at different phosphorylation sites. Results are presented as absorbance (arbitrary value) mean ± SE, n = 4. IR, insulin receptor; IGF-1, insulin-like growth factor 1.

**Figure 5:**
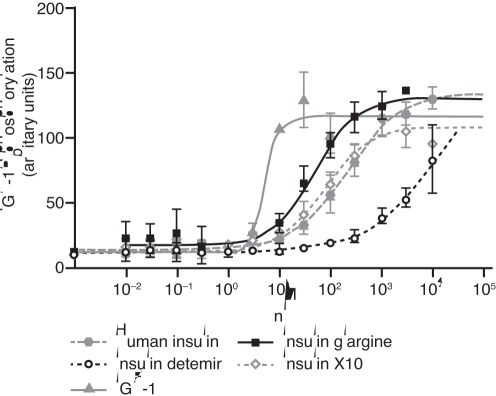
Dose-response curves for activation of the IGF-1 receptor. Data were obtained using BHK cells overexpressing human IGF-1R with immunometric assay for phosphorylated IGF-1R, and are means (±SE) of 2–4 measurements. Results are presented as mean ± SE, n = 2–4. BHK, baby hamster kidney; IGF-1, insulin-like growth factor 1; IGF-1R, insulin-like growth factor 1 receptor.

Data for phosphorylation of the three representative regions of the IR isoforms are shown in Fig. 4 and the calculated relative potencies given in Table 3. Detemir and glargine showed a balanced degree of phosphorylation across the three sites, with relative potencies corresponding to the IR binding affinities. This was also the case for IGF-1, whereas X10 seemed to induce proportionately more phosphorylation of the J and K regions relative to the C region. In agreement with the IR binding data, glargine and detemir each showed balanced activation potency at the two isoforms of the IR. Dose-response curves for activation of IGF-1R by the study ligands relative to human insulin are shown in Fig. 5. Compared to human insulin, the dose-response curve for IGF-1R activation was (as expected) greatly left-shifted for IGF-1 (potency ∼4000%). The curves were also slightly left-shifted for glargine and X10 resulting in relative potencies of ∼480% and ∼250%, respectively. For detemir, the curve was right-shifted, evidence of a lower potency than human insulin (8%) with respect to IGF-1R activation.

**Table 3:** Relative potencies for activation of insulin receptor isoforms A and B at different phosphorylation sites.

	IR-A			IR-B		
	Juxta-membrane	Kinase	C-terminal	Juxta-membrane	Kinase	C-terminal
Human insulin	100	100	100	100	100	100
Insulin X10	409	284	197	638	420	246
Insulin detemir	3.1	4.7	4.2	3.7	4.6	3.6
Insulin glargine	34.0	36.5	40.8	35.2	38.4	39.9
IGF-1	8.0	7.6	5.7	1.1	1.1	0.7

Data are means ± SE, n = 4.

IGF-1, insulin-like growth factor 1.

### Duration of Receptor Activation

**Figure 6:**
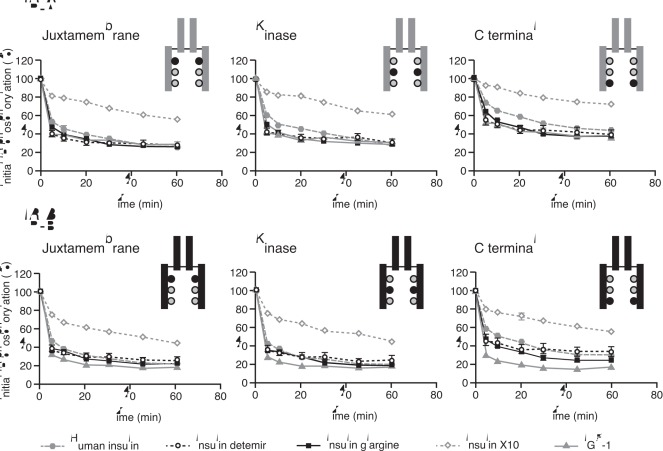
Duration of activation of insulin receptor isoform A and isoform B at different phosphorylation sites. Results are presented as mean ± SE, n = 8. IR, insulin receptor; IGF-1, insulin-like growth factor 1.

**Figure 7:**
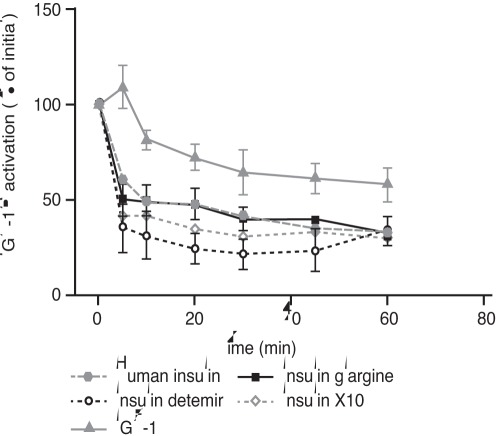
Duration of activation of the IGF-1 receptor. Data are means (±SE) of three measurements. Results are presented as mean ± SE, n = 3. IGF-1, insulin-like growth factor 1.

Data showing the rate of decline of activation (phosphorylation) by ligand for each of the three studied regions of IR-A, IR-B and IGF-1R are presented in Fig. 6 and Fig. 7. The rate of decline of IR phosphorylation was similar for human insulin, detemir and glargine, suggesting that these ligands evoke very similar signalling kinetics at both IR isoforms irrespective of the phosphorylation sites. In contrast, X10 exhibited a slower rate of decline than human insulin for IR phosphorylation on both IR isoforms and at all the studied phosphorylation sites. At the IGF-1R, all the insulin analogues showed a more rapid decline in phosphorylation than IGF-1 itself. At 60 minutes after stimulation with IGF-1, more than 60% of the initial phosphorylation was still present, similar to the observation with X10 at the IR.

### Cell Mitogenicity

**Figure 8:**
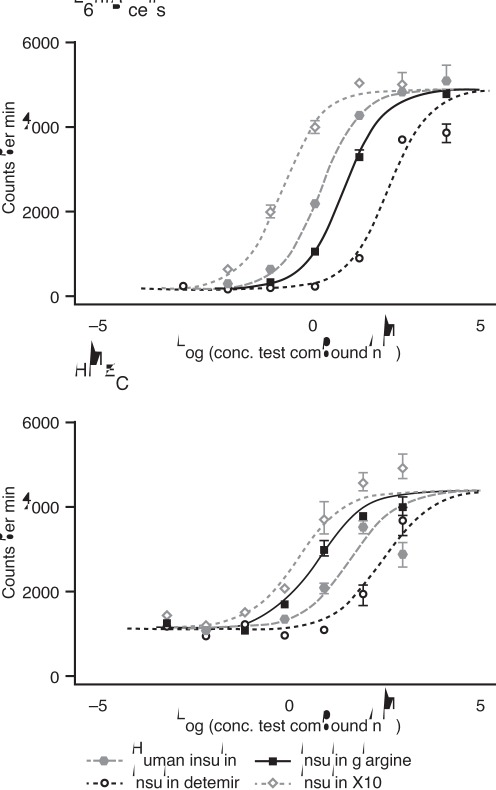
Dose-response curves for mitogenic activity in two cell types (L6-hIR, predominantly expressing IR-A, and HMEC, predominantly expressing IGF-1R). Data were obtained by measurement of ^3^H-thymidine incorporation into DNA. The figure shows representative data. Relative potencies are calculated in table 5 (n = 9–17). IR, insulin receptor; HMEC, human mammary epithelial cells.

Relative proportions of human IR and IGF-1R in each cell line or type are shown in Table 4. Mitogenicity of the studied ligands was assessed by full dose-response curves for ^3^H-thymidine incorporation into DNA as shown in Fig. 8. In L6-hIR cells, which predominantly express insulin receptors (IR-A), X10 showed a leftward shift in the dose-response curve compared to human insulin resulting in a relative potency of 619±61%, while glargine and detemir exhibited rightward shifts leading to relative potencies of 49±9% and 9±2%, respectively (Table 5). The mitogenic potencies measured in the L6-hIR cells therefore reflect the relative IR binding affinities for glargine and detemir, but not for X10, which displays a mitogenic potency in excess of its binding affinity. In the HMEC cell type, which predominantly express IGF-1 receptors, both X10 and glargine showed a substantial leftward shift in their dose-response curves relative to human insulin, which resulted in increased mitogenic potencies of 1098±235% and 650±136%, respectively, whereas detemir again showed a rightward shift in the dose-response curve and therefore a decreased mitogenic potency of 17±3% relative to human insulin (Table 5).

**Table 4:** The specific antibody binding capacity (SABC) for human mammary epithelial cells (HMEC) and L6-hIR cells.

Cell type	Insulin Receptor	IGF-1R	IR:IGF-1R ratio
L6-hIR	204112±22856	N/A	2∶1*
HMEC	1023±168	21313±4279	1∶21

The SABC (average number of antibodies capable of binding to each cell) was measured using either the murine monoclonal antibody 83–7 recognising the human IR or 24–31 recognising the human IGF-1R. Data represent mean (±SD) of at least three independent experiments. No antibody is available that recognises the extracellular domains of the rat IGF-1R, therefore it is not possible to determine the relative number of rat IGF-1R on L6-hIR cells.*unpublished results obtains by Western blot.

IGF-1R, insulin-like growth factor 1 receptor; N/A, not applicable.

**Table 5:** Relative mitogenic potencies in L6-hIR cells and human mammary epithelial cells (HMEC).

	Mitogenic potency relative to human insulin (%)	
	L6-hIR	HMEC
Human insulin	100	100
Insulin X10	617±61	1098±235
Insulin glargine	49±9	650±136
Insulin detemir	9±2	17±3
IGF-1	3±1	4964±1225

Potencies are presented as mean (±SE) from ≥9 independent experiments.

## Discussion

In the present study we have confirmed earlier results [13] showing that detemir has an IGF-1R:IR binding affinity ratio of ≤1 relative to human insulin and that detemir displays a dissociation pattern from the IR, which is similar to that of human insulin. Consequently, the relative mitogenic potency of detemir in cell types predominantly expressing either the IGF-1R (HMEC) or the IR (L6-hIR) is low and corresponds to its IGF-1R and IR affinities. In contrast, X10 and glargine, relative to human insulin, displayed higher IGF-1R affinities resulting in relative IGF-1R:IR binding ratios >1 (versus human insulin), a finding which is in agreement with previous results [13], [23]. In addition, increased phosphorylation of the IGF-1R after stimulation with glargine has been shown [24], [25]. The increased IGF-1R:IR binding affinity ratio translates into an increased mitogenic potency for X10 and glargine in cells which express many IGF-1 receptors. Even though controversies regarding the mitogenic potency of glargine can be found in literature (reviewed in Hansen [15]), from the data presented here as well as recent data from other groups [23]–[26] it now seems safe to conclude that glargine as well as other insulin analogues with increased relative IGF-1R:IR binding ratios will exhibit an increased mitogenic potency relative to human insulin in cells expressing many IGF-1 receptors. The relative IGF-1R:IR binding ratio for IGF-1 itself is much higher than for X10 or glargine, and it could be argued that compared to IGF-1, insulin analogues would only have a negligible effect on the IGF-1R. However, it has to be taken into account that IGF-1 is bound to IGF-1 binding proteins, and therefore the free fraction of IGF-1 is only a small fraction of the total concentration. Both Sommerfeld et al. [23] and Varewijck et al. [25] found only a 30-fold difference in EC_50_ for the activation of IGF-1R after stimulation with IGF-1R and glargine. Thus the difference between IGF-1 and glargine in EC50 for activation of IGF-1R might not be very large, which is also supported by the present study.

Equilibrium binding studies have revealed that insulin detemir can displace ^125^I-insulin from the receptors in a identical manner to human insulin albeit with a lower potency. However, because of the albumin binding properties of insulin detemir, the EC_50_ estimate will depend on the prevailing concentration of albumin [27]. As human insulin and other insulin analogues with no lipid side chains attached bind negligibly to albumin, the EC_50_ estimates do not depend on the albumin concentration in a given assay. Thus, the degree of right-shift for insulin detemir’s concentration–response curve increases with increasing albumin concentrations.

The amount of albumin used in the different assays is determined partly by historical reasons and partly by the requirement of assays [13]. Several of the assays used in this paper can be performed at conditions where no albumin or serum is added and as such the potencies measured in these assays can be viewed as reflecting the ‘albumin-free’ potency of insulin detemir. In the absence of albumin, the relative potencies of insulin detemir were estimated to be 17–26%, whereas values in the presence of albumin were in the range 3–6%. This decrease in relative potency with increase in the albumin concentration is in accordance with previous observations [27].

With respect to mitogenic potencies in L6-hIR and HME cells, the potencies were estimated to be 9% and 17% relative to human insulin, respectively. With HMEC, it was possible to perform the assay in total absence of albumin, whereas 0.1% serum was needed in the case of L6-hIR. The albumin content in serum is low, but resulted in a slight decrease in potency in L6-hIR cells compared to HMEC, nevertheless.

Recent studies have suggested that detemir displays an increased IGF-1 like activity and mitogenic potency compared with human insulin: Weinstein et al. [28] examined a number of cancer cells and concluded that detemir along with several other insulin analogues exhibited *in vitro* proliferative and anti-apoptotic activities compared to human insulin. However, the responses obtained in that study were very modest and in dose-response experiments the authors failed to show any significant effects of insulin and IGF-1, making conclusions somewhat dubious. In addition, this study has been criticised for inconsistency in the experimental methodology applied for the different ligands [29]. Sciacca et al. [30] also reported a mitogenic potency of detemir on par with that of glargine. However, the authors did not perform dose-response experiments for the measurement of mitogenic potencies and the observed responses were *very* modest. This was reflected by the lack of effect of the positive control X10 in cells expressing insulin receptors. When comparing insulin analogues in cellular systems it is necessary to perform full dose-response curves and to optimise the assay system to give a proper response (at least a two-fold difference in maximal response) in order to obtain meaningful comparison between analogues [7].

In addition, Sciacca et al. [30] also reported an increased IGF-1R binding affinity for detemir compared with human insulin. In fact, they reported a *higher* IGF-1R affinity for detemir than for X10. This is in sharp contrast to our present findings as well as previous studies [13], [25], which reported significantly reduced relative IGF-1R binding and activation for detemir when performing full dose-response curves. The explanation for this discrepancy must relate to methodological differences. It is possible that the use of Scatchard plots for analysis of binding affinities as applied by Sciacca et al. [30] could be an explanation, since this linear regression method distorts the experimental error. Scatchard transformation also violates the assumptions of linear regression and is therefore only useful for displaying binding data, which should always be analysed by non-linear regression. Nevertheless, on the basis of the present and earlier studies [13], [25] it seems safe to conclude that the relative IGF-1R binding affinity, IGF-1R activation and mitogenic potency of detemir is significantly lower than that of human insulin and in the same range as the relative binding affinity for the IR.

No major differences between the three IR phosphorylation sites examined were seen for detemir or glargine. Thus, the relative potencies were comparable across the three phosphorylation sites, which was also the case for IGF-1. In contrast, there seems to be a preferential phosphorylation of the Juxtamembrane site and, to a lesser degree, the kinase domain phosphorylation sites after stimulation with X10. Potentially, this could be a unique and interesting feature with super-mitogenic insulin analogues mediating an increased mitogenic potency via the IR. However, more experiments are needed to further elaborate on these findings, for example, using a panel of different insulin analogues with variable mitogenic potencies. Furthermore, various periods of stimulation are also required in order to fully explore this possibility, since it is well-known that insulin analogues with high receptor affinities often show altered receptor-binding kinetics.

It has been speculated that an increase in the mitogenic properties of an insulin analogue could alternatively (or additionally) reflect a binding preference for the shorter IR-A isoform of the IR relative to the longer IR-B isoform [30], [31]. This hypothesis derives from the observation that IR-A has high affinity for binding insulin-like growth factor 2 (IGF-2) and is extensively expressed in foetal tissue, where it mediates growth responses. IR-A expression is also associated with undifferentiated cells, and over-expression occurs in some cancer cells. This raises the possibility that this IR isoform may be relevant for the mitogenesis of cancer cells [31]. In their recent work, Sciacca et al. [30] reported that detemir displayed a 13-fold difference in the affinity between IR-B and IR-A in favour of IR-A. This was in sharp contrast to the present study, where we find a balanced IR isoform binding, as well as IR isoform activation for detemir, glargine and X10. We have studied several hundred insulin analogues including the commercially available insulin analogues and X10 using both isoforms of the IR [20], [32] and have never identified an insulin analogue with as much as a 13-fold difference in IR isoform affinity. The discrepancy between our finding and the observation in IR isoform affinity observed by Sciacca et al. [30] is therefore most likely due to the above mentioned methodological differences.

A methodologically challenging aspect that has not been investigated in great detail is the question of hybrid receptors formed between the IR and IGF-1R and their importance for mediating mitogenic responses. Evidence suggests that hybrid receptors bind insulin with the same affinity as IGF-1R [17] and it could be speculated that the increased mitogenic potency of analogues with increased affinity for the IGF-1R in fact was mediated via IR/IGF-1R hybrid receptors. Some cancer cells express higher levels of IR-A and IGF-1R and hence form Hybrid-A receptors [31]. In the light of evidence that Hybrid-A has a higher affinity for human insulin than Hybrid-B [33], it could be speculated that insulins with high affinities for Hybrid-A could drive cancer growth through activation of Hybrid-A. However, in previous studies we did not find Hybrid-A to bind insulin with high affinity; in contrast we found that the two splice variants of hybrid receptors bind insulin with similar low affinity [17]. In this study we included X10, glargine and detemir in order to test their affinities for hybrid receptors and explore whether these differ between the two splice variants of hybrid receptors. We did not find significant differences in binding between the two hybrid receptor splice variants for any of the analogues tested in this study. However, we did find that X10 and glargine, which had relatively higher affinity for IGF-1R, also bound to both hybrid receptors with three-fold higher affinities than human insulin. In contrast, detemir bound both hybrid receptors with a four-fold decreased affinity compared to human insulin. Compared to the high affinity IGF-1 binding to hybrid receptors, the tested analogues bound at a level of 0.3–5% affinity. The selectivity between IGF-1 and the tested analogues for hybrid receptors were at the same level as for their cognate homodimer receptors.

The cell lines employed in mitogenicity testing contained different levels of IRs and IGF-1Rs (Table 4). It is assumed that hybrid receptors formed between IRs and IGF-1Rs occurs in a random fashion in the ER. If this is the case then the level of IR in hybrid receptors can be calculated by the mathematical formula 1/([IR]/[IGF-1R]+1) [34]. Then HMEC cells would have 95% of their IR bound in hybrid receptors. The level of insulin-responsive homodimer receptors would then be significantly decreased compared to the expected level in hybrid receptor formation. The two insulin analogues, X10 and glargine, were the only insulin analogues we have tested that had a higher affinity to hybrid receptors compared to human insulin. It can be speculated that some of the mitogenicity of these two analogues may be through activation of hybrid receptors, as their affinity was comparable to homodimer IGF-1R affinity.

We have previously reported that sustained signalling from the IR correlated with an increased mitogenic potency of an insulin analogue [14]. In this study, we have now extended our previous finding by examining three different IR phosphorylation sites, the two isoforms and also the extent of signalling from the IGF-1R. The overall conclusion from these studies is that only X10 displays sustained signalling from the IR and only IGF-1 displays sustained signalling from the IGF-1R. For each ligand, no major differences were seen between the different phosphorylation sites. The only exception was that C-terminal phosphorylation seems to decline a little faster after stimulation with IGF-1. By comparing the duration of signalling from IR-A with that of IR-B it seems that the signal declines slightly faster from IR-B, which was a general feature observed for all ligands and all phosphorylation sites. The significance of this phenomenon remains to be elucidated.

The data presented herein clearly show that insulin analogues with an increased affinity for the IGF-1R also have an increased mitogenic potency in cells expressing many IGF-1 receptors. Thus, X10 and glargine both display increased mitogenic potencies in HMEC cells, which express approximately 21-fold more IGF-1 than insulin receptors. X10, but not glargine, also displays an increased mitogenic potency in cells predominantly expressing IR. Therefore, it seems clear that glargine mediates its increased mitogenic potency through the IGF-1R and not through the IR. This conclusion is also supported by studies by Shukla et al. [26] where IGF-1R receptor levels were knocked down by siRNA technique and earlier studies by Eckardt et al. [35], in which IGF-1R levels were manipulated by clone selection procedures.

X10 displays an increased relative mitogenic potency in both cell types employed in this study, which predominantly express either the IR (L6-hIR cells) or the IGF-1R (HMEC); thus X10 seems to be able to evoke an increased mitogenic response through both mechanisms shown in Fig. 1. As previously shown [14], X10 displays a greatly sustained signalling from the IR and we have now extended that observation to include both receptor isoforms and several IR phosphorylation sites, while we also have excluded the possibility that X10 is able to induce sustained signalling from the IGF-1R. Finally, we have found indications for a preferential phosphorylation of the most N-terminal phosphorylation sites after stimulation with X10. Further research is needed in order to dissect which of these mechanisms is the most important factor driving the increased mitogenic potency of X10 in cells via the IR. The finding that substantial differences exist between cell types underscores the need for proper characterisation of the cell systems applied for mitogenicity studies. The receptor number and effects of native ligands and the positive control X10 is especially important.

At present, it is unknown which of the two main mechanisms, or a combination thereof (depicted in Fig. 1), accounted for the dose-dependent increase in the incidence of mammary tumours in female Sprague-Dawley rats observed after stimulation with X10 [8]. There is a strong need for improved animal models in order to test the tumour-promoting effects of insulin analogues. Ideally, such a model should be a diabetic and/or an insulin resistant model, since this would avoid the very low glucose levels seen in traditional animal toxicological studies and therefore resemble the clinical situation more closely. Once available, such models would allow a more detailed correlation between *in vitro* molecular characteristics and *in vivo* tumour promoting effects of insulin and insulin analogues.

In summary, our data show that neither glargine nor detemir differ from human insulin in their relative affinities for the two IR isoforms (either in homodimer form or as hybrid receptors with IGF-1R), or in their ability to stimulate the three studied IR phosphorylation sites or in signalling kinetics; neither analogue has an increased mitogenic effect in cells that express predominantly IR. X10 and glargine do, however, display an increased relative binding affinity for the IGF-1 receptor compared to the insulin receptor (versus human insulin) and consequently exhibit increased mitogenic activities in cells predominantly expressing IGF-1R. X10 displays an increase in the relative IGF-1R:IR affinity ratio as well as prolonged IR signalling kinetics and is more mitogenic than human insulin in both IR and IGF-1R-expressing cells. Importantly, none of the molecular data presented in this paper give rise to any safety concern with detemir.
